# Development and validation of a collagen signature to predict central lymph node metastasis in papillary thyroid cancer

**DOI:** 10.3389/fendo.2025.1691788

**Published:** 2025-11-28

**Authors:** Weisheng Chen, Junna Ge, Tingyue Luo, Shumin Dong, Wei Jiang, Cangui Wu, Buning Ye, Dongling Zhang, Wanying He, Jun Yan, Shangtong Lei

**Affiliations:** 1 Department of General Surgery, Nanfang Hospital, Southern Medical University, Guangzhou, Guangdong, China; 2 Chronic Airways Diseases Laboratory, Department of Respiratory and Critical Care Medicine, Nanfang Hospital, Southern Medical University, Guangzhou, Guangdong, China; 3 Department of Thyroid Surgery, The First Affiliated Hospital of Zhengzhou University, Zhengzhou, China

**Keywords:** collagen signature, tumor microenvironment, papillary thyroid cancer, predictive model, lymph node metastasis

## Abstract

**Background:**

Current clinicopathological risk factors lack the precision necessary for accurate prediction of central lymph node metastasis (CLNM) in patients with papillary thyroid cancer (PTC). Structural remodeling of the tumor microenvironment (TME), particularly collagen organization, may play a pivotal role in metastatic dissemination.

**Objective:**

The objective of this study was to develop a collagen signature within the TME to predict CLNM in PTC and validate that the new model incorporating it into the assessment alongside clinicopathological risk factors would enhance the predictive accuracy.

**Methods:**

In this retrospective study, we included 350 patients with classic PTC, all of whom underwent thyroidectomy with prophylactic central lymph node dissection. The cases were randomly assigned to a training cohort and a testing cohort with a 6:4 ratio. A total of 142 collagen features in the TME were extracted from second harmonic generation images of tumor specimens. We constructed a collagen signature using a least absolute shrinkage and selection operator (LASSO) regression model. Multivariate logistic regression was used to integrate the signature with clinicopathological variables and construct a nomogram.

**Results:**

The predictive ability of collagen signature was also validated by AUC of 0.821 in training cohort and AUC of 0.793 in testing cohort. The collagen signature remained an independent predictor after adjustment for tumor size, capsular invasion, and tumor location in the multivariate analysis. Furthermore, the integrated model showed superior predictive performance compared to the clinicopathological model alone (0.842 vs. 0.679, *p* < 0.001). Decision curve analysis confirmed higher net clinical benefit across a wide range of thresholds.

**Conclusions:**

The collagen signature within the TME represents a promising new biomarker that can effectively predict CLNM in PTC patients, potentially improving clinical decision-making and patient management.

## Introduction

Papillary thyroid carcinoma (PTC) is the most common endocrine malignancy and frequently exhibits central lymph node metastasis (CLNM), with reported rates ranging from 40% to 90% (1–5). Accurate assessment of CLNM is essential for surgical planning, as inadequate or excessive lymph node dissection can respectively increase recurrence risk or operative morbidity (6). However, preoperative imaging, particularly ultrasound, often fails to detect occult metastases due to the deep anatomical location of central lymph nodes. Traditional risk stratification factors including age, tumor size, extrathyroidal extension, and histological subtypes, provide limited predictive accuracy, which underscores the need for novel biomarkers to guide individualized management (6–9).

Recent advances in molecular profiling, including *BRAF* mutation testing and circulating RNA signatures, have shown potential in predicting lymph node metastasis (10, 11). Nevertheless, these methods require further validation before they can be widely implemented in clinical practice. Additionally, collagen, the main component of the tumor microenvironment (TME), plays a critical role in tumor development and progression (12), and has emerged as an important predictive and prognostic biomarker in various malignancies, including gastric, breast, and colon cancers (13–16).

Second harmonic generation (SHG) imaging is a label-free optical technique that visualizes and quantifies collagen architecture (17, 18).This quantitative method provides valuable information about collagen in complex TME and has been employed successfully in diagnosing several diseases (19–21).

In this study, we utilized SHG imaging to quantify collagen changes in the TME of PTC and investigated the predictive utility of the resulting collagen signature for CLNM. This approach offers a powerful means to assess collagen characteristics and may serve as a robust predictive tool for lymph node metastasis in PTC patients.

## Materials and methods

### Patient selection and study design

This study was conducted at the Department of General Surgery, Nanfang Hospital of Southern Medical University, and included patients treated between January 2018 and June 2019. The inclusion criteria were as follows: age ≥18 years, histologically confirmed classic papillary thyroid cancer (PTC) after radical thyroid surgery, and the availability of both clinicopathological data and pathological specimens. Patients were excluded if they had previously undergone thyroid therapy, had a history of other malignant tumors, or were diagnosed with concurrent Hashimoto’s thyroiditis. In accordance with the thyroid cancer treatment guidelines of the Chinese Society of Clinical Oncology, all patients underwent either lobectomy or total thyroidectomy, accompanied by prophylactic central lymph node dissection (22, 23). The following clinicopathological characteristics were collected for analysis: gender, age at the time of surgery, body mass index (BMI), maximum tumor size, the number of tumor lesions, tumor location within the thyroid gland, the status of thyroid capsular invasion (TCI), the status of BRAF V600E mutation and ultrasound features. Maximum tumor size was measured as the largest diameter of the tumor in millimeters. The cutoff for tumor size (>10 mm) followed the 2015 ATA guideline definition of clinically significant PTC, applied *a priori* before statistical modeling. Tumor location was classified as upper versus middle-inferior according to craniocaudal thirds of the thyroid lobe. TCI was determined by histopathological examination. BRAF V600E mutation status was determined by PCR amplification and Sanger sequencing of exon 15 from formalin-fixed paraffin-embedded specimens. Preoperative ultrasound characteristics including microcalcification, margin definition, echogenicity, and shape ratio (height/width), were independently reviewed by two blinded radiologists.

The included cases were randomly assigned to a training cohort and a testing cohort with a 6:4 ratio. This study was approved by the Institutional Review Board of Nanfang Hospital of Southern Medical University (Approval No. NEFC-2021-217), and the research was conducted in line with the principles of the Declaration of Helsinki.

### Selection of the regions of interest, multiphoton image acquisition, and collagen feature extraction

**Figure 1:**
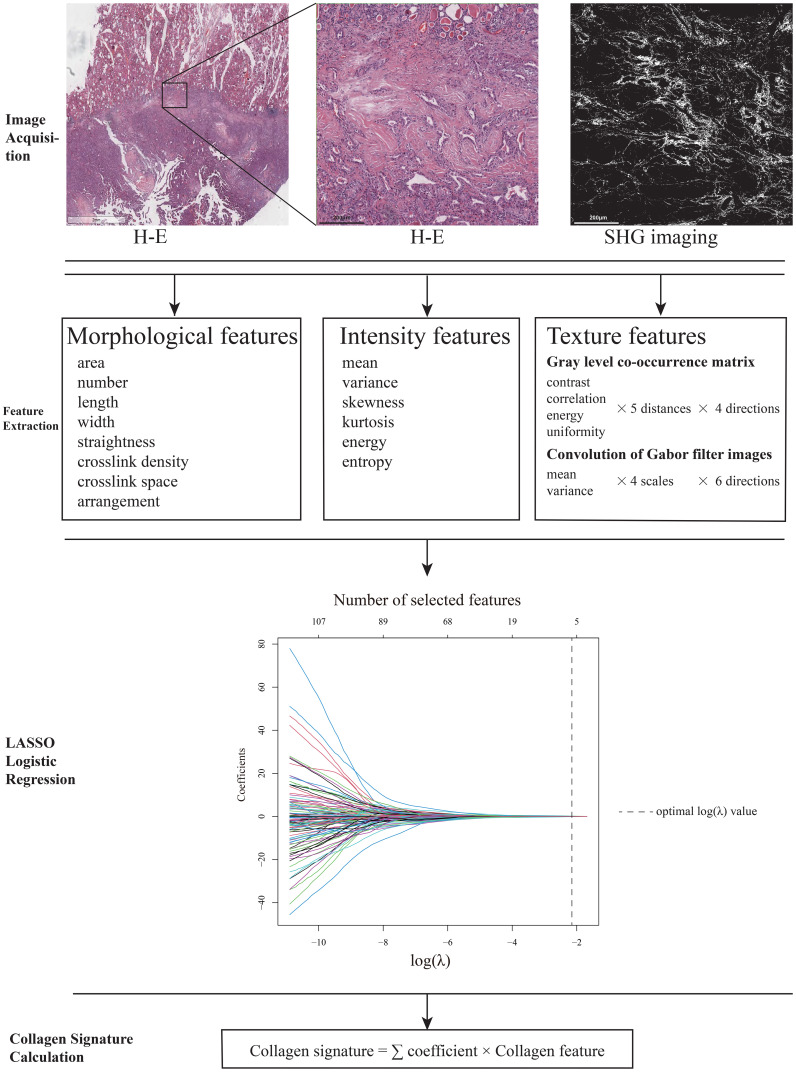
Study protocol. Two pathologists independently identified the region of the invasive margin of the thyroid cancer sample, and five regions of interest with fields of view of 1000 μm×1000 μm per specimen that were equidistantly spread throughout the invasive margin were selected. An unstained serial section was used for multiphoton imaging, and the SHG images were transformed to gray-scale for feature extraction. LASSO logistic regression was conducted to select the collagen features with the greatest prognostic value. By combining the selected collagen features and the corresponding coefficients, the collagen signature of each patient was calculated with a standard linear formula. All histological and SHG images include scale bars.

We obtained formalin-fixed, paraffin-embedded (FFPE) specimens from all patients; specimens were serially sectioned and stained with hematoxylin-eosin (H-E). Two pathologists who were blinded to the status of lymph node metastasis of the patients independently identified the region of the invasive margin of the thyroid cancer sample with a microscope at 200× magnification. Discrepancies were resolved through consensus with a senior pathologist. To ensure an accurate representation of each sample, five regions of interest (ROIs) were chosen along the invasive margin. These ROIs were equidistantly spread and had a field of view (FOV) of 1000 μm × 1000 μm per specimen. An unstained serial section was used for multiphoton imaging with a 20× objective (**Figure 1**). The multiphoton imaging system has been previously described (14, 24). The excitation wavelength used in this study was 800 nm.

Collagen features were extracted from SHG images using MATLAB 2015b. A total of 142 features were quantified, including morphological (n=8), intensity (n=6), and textural (n=128) parameters as previously reported (19, 20, 25). The morphological features included the following information about the collagen fibers: the area, number, length, width, straightness, crosslink density, crosslink space, and arrangement. The intensity features included the mean, variance, skewness, kurtosis, energy, and entropy of the collagen fibers. The texture features included the contrast, correlation, energy, and uniformity of the gray level co-occurrence matrix (GLCM) at five different pixel distances and in four different directions, and the mean and variance of the convolution of Gabor filter images at four scales and in six directions. Patients’ feature values were calculated by averaging the measurements of the five ROIs. All features were standardized using Z-score normalization based on the distribution of the training cohort, and these parameters were applied directly to the testing cohort to prevent data leakage.

### Development and validation of the prediction model

Least-absolute shrinkage and selection operator (LASSO) logistic regression was conducted to select the collagen features in the training cohort with the most predictive value, using R (version 4.3.3) with the “glmnet” package in this study (26, 27). Five-fold cross-validation was used to determine the optimal tuning parameter λ. The status of CLNM of each patient was used in the LASSO model. By combining selected collagen features and LASSO logistic regression coefficients, the collagen signature of each patient was calculated with a standard linear formula. The area under the curve (AUC) of the receiver-operating characteristic (ROC) curve of the collagen signature was measured.

Univariate and multivariate logistic regression analyses were performed to evaluate the association between the collagen signature, seven clinicopathological factors, and CLNM in both cohorts. The odd ratio (OR) and 95% confidence interval (95% CI) were reported, with statistical significance defined as *p* < 0.05.

A nomogram integrating the collagen signature with independent clinicopathological predictors was constructed in the training cohort. Discrimination was assessed using the concordance index (C-index) with bootstrap validation. ROC curves and DeLong’s test were used to compare model performance across cohorts. Decision curve analysis (DCA) was performed to assess clinical utility. The models could include numerical variables alone (for example collagen signature), or categorical variables alone (for example TCI status) and both of them (for example new model) (28, 29). The clinicopathological model included only clinicopathological risk factors in the multivariate logistic regression.

To evaluate the clinical utility of our predictive model, we performed decision curve analysis (DCA). This method compares the net benefit of the model to that of standard treatment strategies across a range of threshold probabilities.

### Subset analysis incorporating BRAF mutation and ultrasound features

A subset analysis was performed in 120 patients for whom both BRAF mutation and preoperative ultrasound data were available. The predictive performance of the new model, BRAF mutation, ultrasound features, were assessed using AUC with 95% confidence intervals. Model comparisons were conducted using the DeLong’s test.

### Statistical analysis

All feature preprocessing, including aggregation, normalization parameter derivation, and LASSO feature selection with λ tuning, was conducted exclusively in the training cohort. The final coefficients were fixed and directly applied to the standardized testing cohort without refitting to avoid data leakage. A detailed workflow is provided in **Supplementary Figure 1**. ROC curve analysis was performed using the “pROC” package in R. AUCs with 95% CIs were computed using 1000 bootstrap resamples. Categorical variables were presented as frequencies and percentages. Chi-square tests (or Fisher’s exact tests, if applicable) were used to compare the differences between categorical variables. Statistical significance was determined by a *p*-value of less than 0.05. All statistical analyses were performed using SPSS for Windows, version 22.0 (SPSS, Chicago, IL, USA).

## Results

### Patients and clinicopathological characteristics

A total of 210 and 140 patients were randomly assigned to the training and testing cohorts, respectively. The CLNM rates in the training and testing cohorts were 41.0% and 42.1%, respectively (**Table 1**). The clinicopathological characteristics of the two cohorts showed no statistically significant differences.

**Table 1:** Clinical characteristics of the patients in the training and testing cohorts.

Parameter	Training cohort		Testing cohort		p
	N	%	N	%	
Number of patients	210	100.0	140	100.0	
Positive LNM	86	41.0	59	42.1	0.825
Age (years)					0.914
≤50	167	79.5	112	80.0	
≤50	43	20.5	28	20.0	
Gender					0.625
Male	83	39.5	59	42.1	
Female	127	60.5	81	57.9	
BMI					0.429
≤23	120	57.1	74	52.9	
>23	90	42.9	66	47.1	
Location					0.298
Upper portion	51	24.3	41	29.3	
Middle-inferior portion	159	75.7	99	70.7	
Multifocality(US)					0.651
Negative	159	75.7	103	73.6	
Positive	51	24.3	37	26.4	
Maximum tumor size					0.540
≤1cm	115	54.8	72	51.4	
>1cm	95	45.2	68	48.6	
Thyroid capsular invasion					0.081
Negative	100	47.6	80	57.1	
Positive	110	52.4	60	42.9	

### Collagen signature construction

The procedure for the construction of the LASSO logistic regression model was presented in **Figure 1**. The coefficient profiles of the 142 collagen features in thyroid cancer were extracted from the LASSO regression. The optimal value log(λ) was -2.987 and the final model selected the 5 most powerful predictors from among all collagen features, which were the collagen crosslink density, collagen arrangement, mean of collagen intensity, contrast of 45° GLCM of the collagen fibers at direction 2, variance convolution of Gabor filter images on scale 2 at direction 5. Furthermore, the collagen signature of each sample was calculated with the linear formula. (**Supplementary Formula 1**).

### Prediction model for CLNM

**Figure 2:**
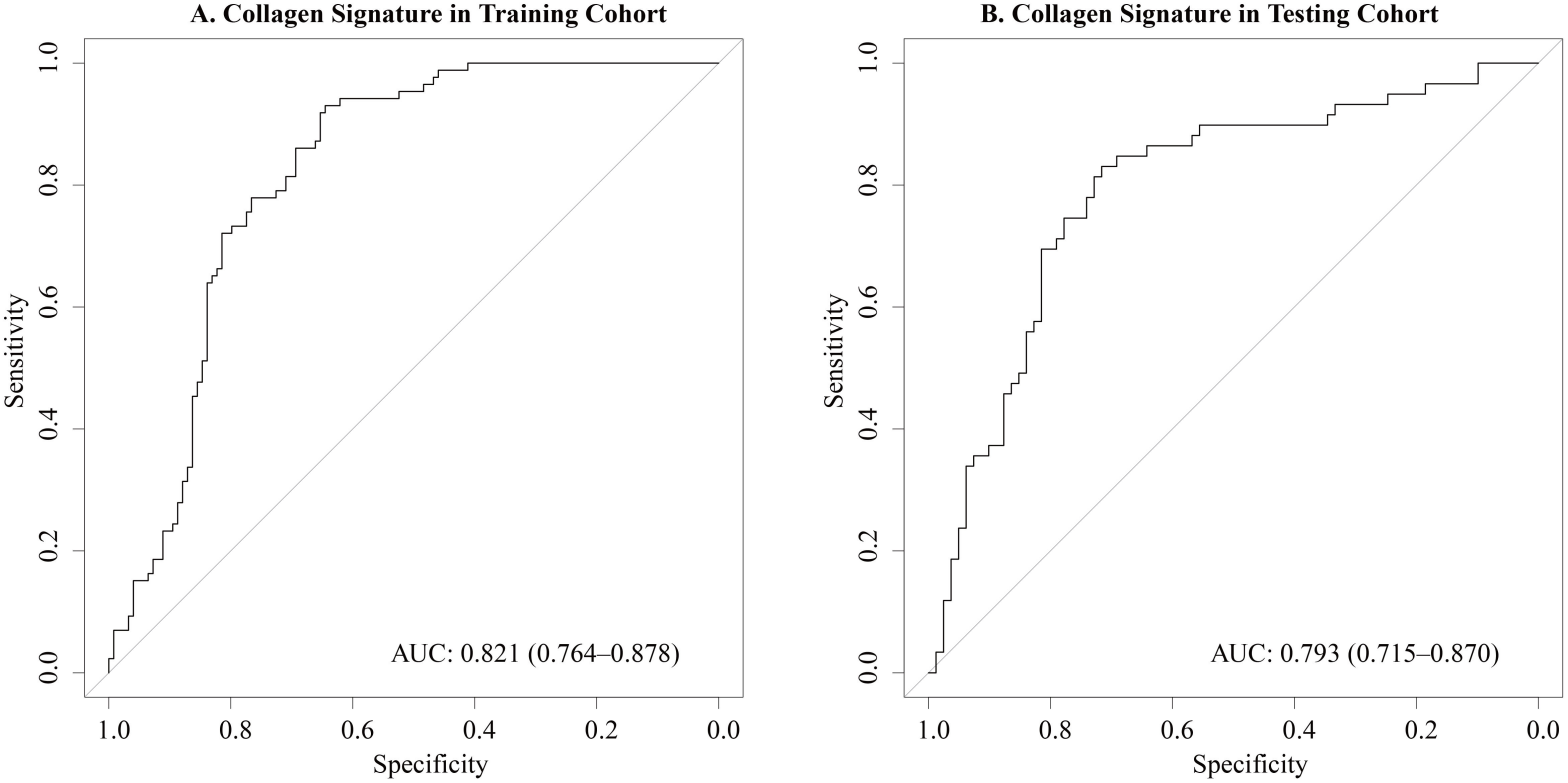
Predictive performance of the collagen signature in the training and testing cohorts. **(A)** The collagen signature predicted the CLNM risk with AUC of 0.821 in the training cohort. **(B)** The collagen signature predicted the CLNM risk with AUC of 0.793 in the testing cohort.

**Figure 2** illustrates the ROC curves of the collagen signature in the training and testing cohorts. The collagen signature had a considerable predictive power, with AUCs of 0.821 (95% CI, 0.764-0.878) in the training cohort, and 0.793 (95% CI, 0.715-0.870) in the testing cohort, respectively. Univariate analyses demonstrated the collagen signature and tumor size, tumor location and TCI were significantly associated with CLNM (*p* < 0.001, *p* = 0.011, *p* < 0.001, and *p* = 0.002, in the training cohort; *p* < 0.001, *p* = 0.020, *p=*0.013, and *p* = 0.021, in the testing cohort; respectively, see in **Supplementary Table 1**). Multivariate analyses (**Table 2**) confirmed that the collagen signature, tumor size, tumor location, and TCI remained independent predictors of CLNM in both cohorts.

**Table 2:** Multivariate Logistic regression analysis of the association of the collagen signature and clinical characteristics with CLNM in the training and testing cohort.

Variables	Training cohort		Testing cohort	
	OR (95% CI)	p	OR (95% CI)	p
Collagen signature	5.263(3.111-8.903)	<0.001	6.609(3.115-14.020)	<0.001
Location (middle-inferior vs. upper portion)	0.381(0.161-0.904)	0.029	0.360(0.136-0.949)	0.039
Maximum tumor size(cm) (≤1 vs ≤1)	3.028(1.501-6.108)	0.002	3.529(1.510-8.245)	0.004
Thyroid capsular invasion (negative vs. positive)	2.227(1.106-4.487)	0.025	2.754(1.178-6.439)	0.019

**Figure 3:**
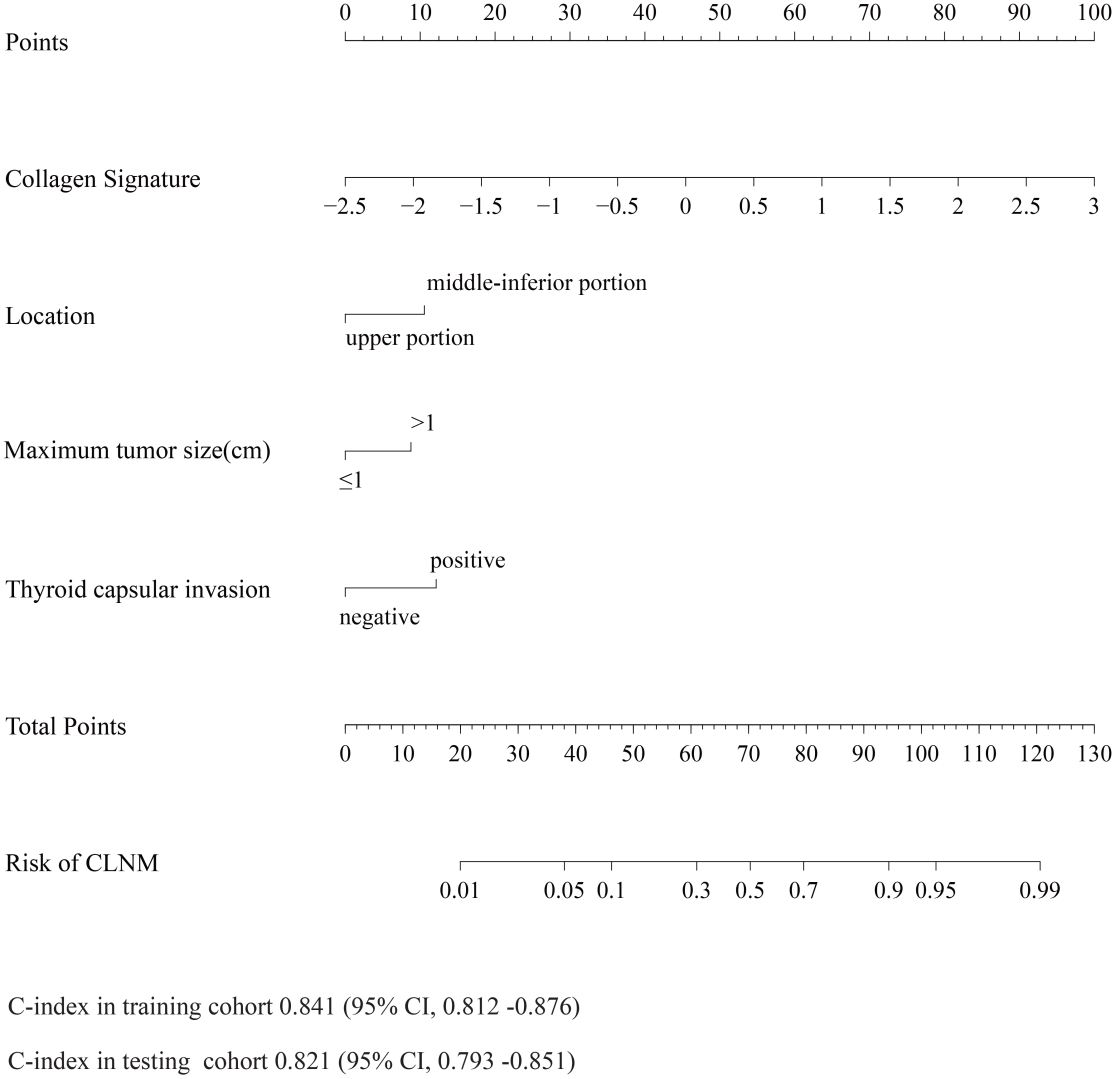
Nomogram of the new model for the prediction of CLNM. The C-indexes of the nomograms of CLNM were 0.841 (95% CI, 0.812 -0.876), 0.821 (95% CI, 0.793 -0.851) in the training and testing cohort, respectively.

**Figure 4:**
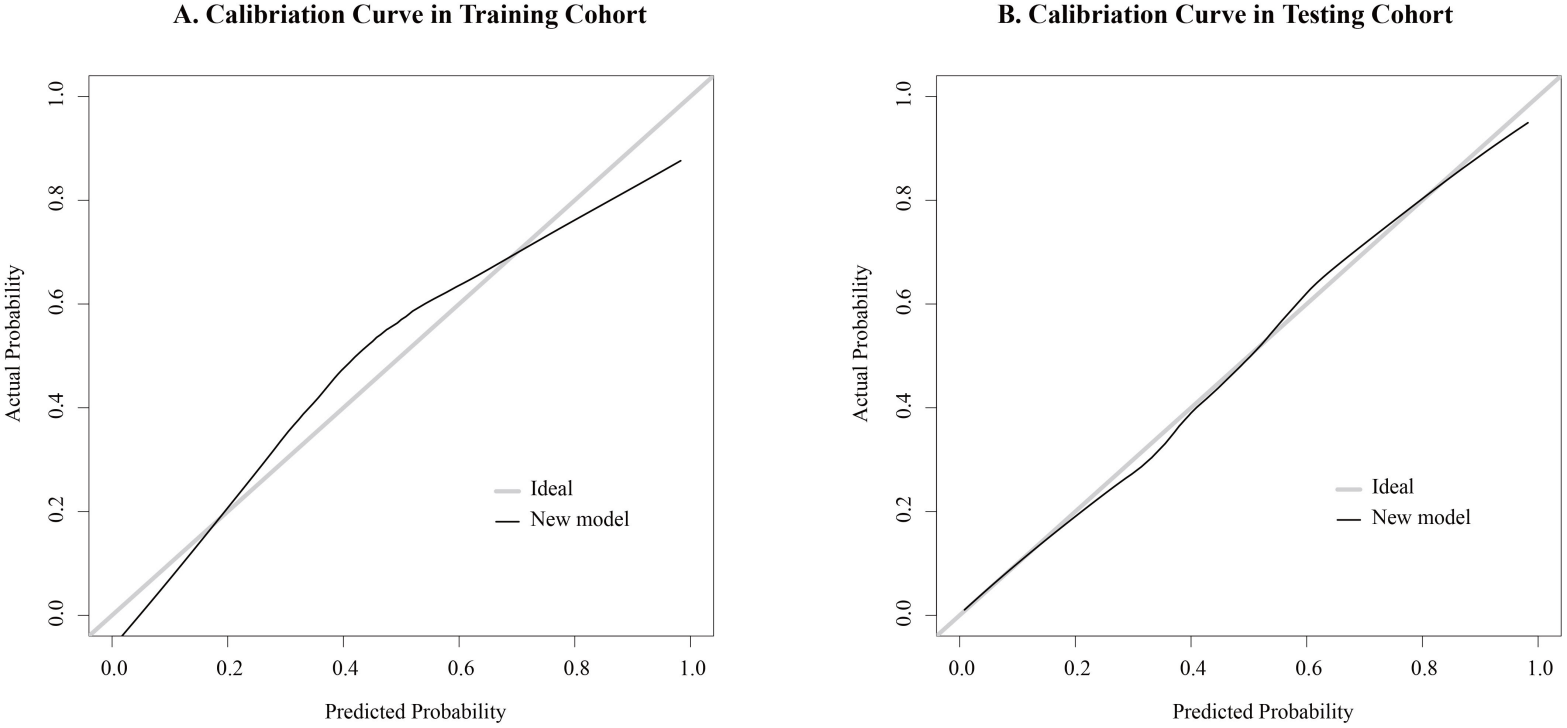
Calibration curve analysis of the new model for the prediction of CLNM. **(A)** The new model demonstrated satisfactory calibration in the training cohort. **(B)** The new model demonstrated satisfactory calibration in the testing cohort.

**Figure 5:**
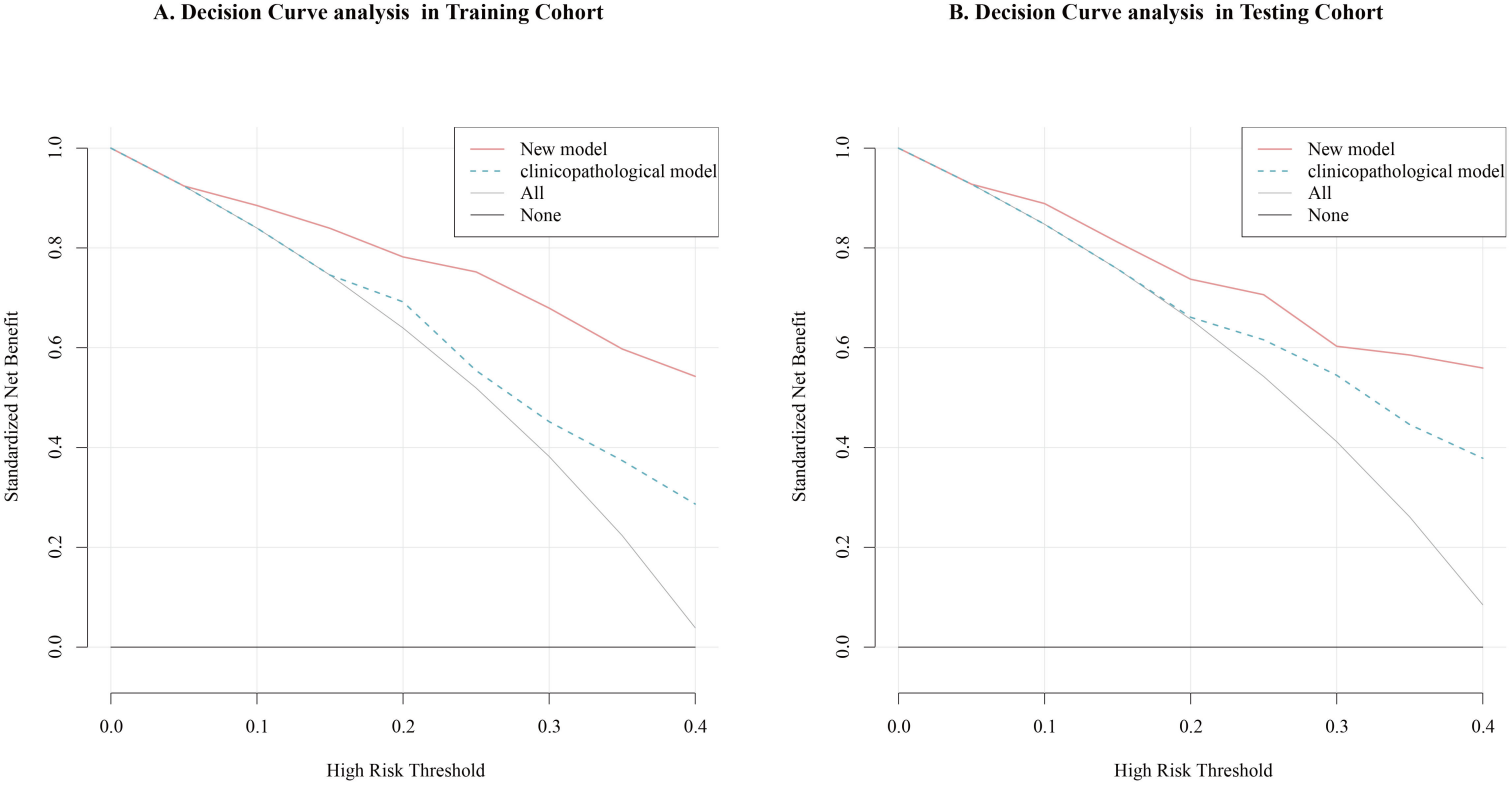
Decision curve analysis of the new model and clinicopathological model for the prediction of CLNM. **(A)** The new model offered a higher net benefit compared to ‘treat-all’, ‘treat-none’, and clinicopathological model strategies across a broad spectrum of threshold probabilities in the training cohort. **(B)** The new model offered a higher net benefit compared to ‘treat-all’, ‘treat-none’, and clinicopathological model strategies across a broad spectrum of threshold probabilities in the testing cohort. The new model combined the collagen signature and clinicopathological risk factors and the clinicopathological model included only clinicopathological risk factors.

The nomogram for the prediction of CLNM was generated on the basis of the collagen signature, tumor size, tumor location and TCI status (**Figure 3**). The C-indexes of the nomograms of CLNM were 0.841 (95% CI, 0.812 -0.876) in the training cohort and 0.821 (95% CI, 0.793 -0.851) in the testing cohort. The nomogram demonstrated satisfactory discrimination and calibration in the two cohorts (**Figure 4**). The decision curve analysis showed that the new model provided greater net benefit across a wide range of threshold probabilities compared with the treat-all, treat-none, and clinicopathological strategies. (**Figure 5**).

**Figure 6:**
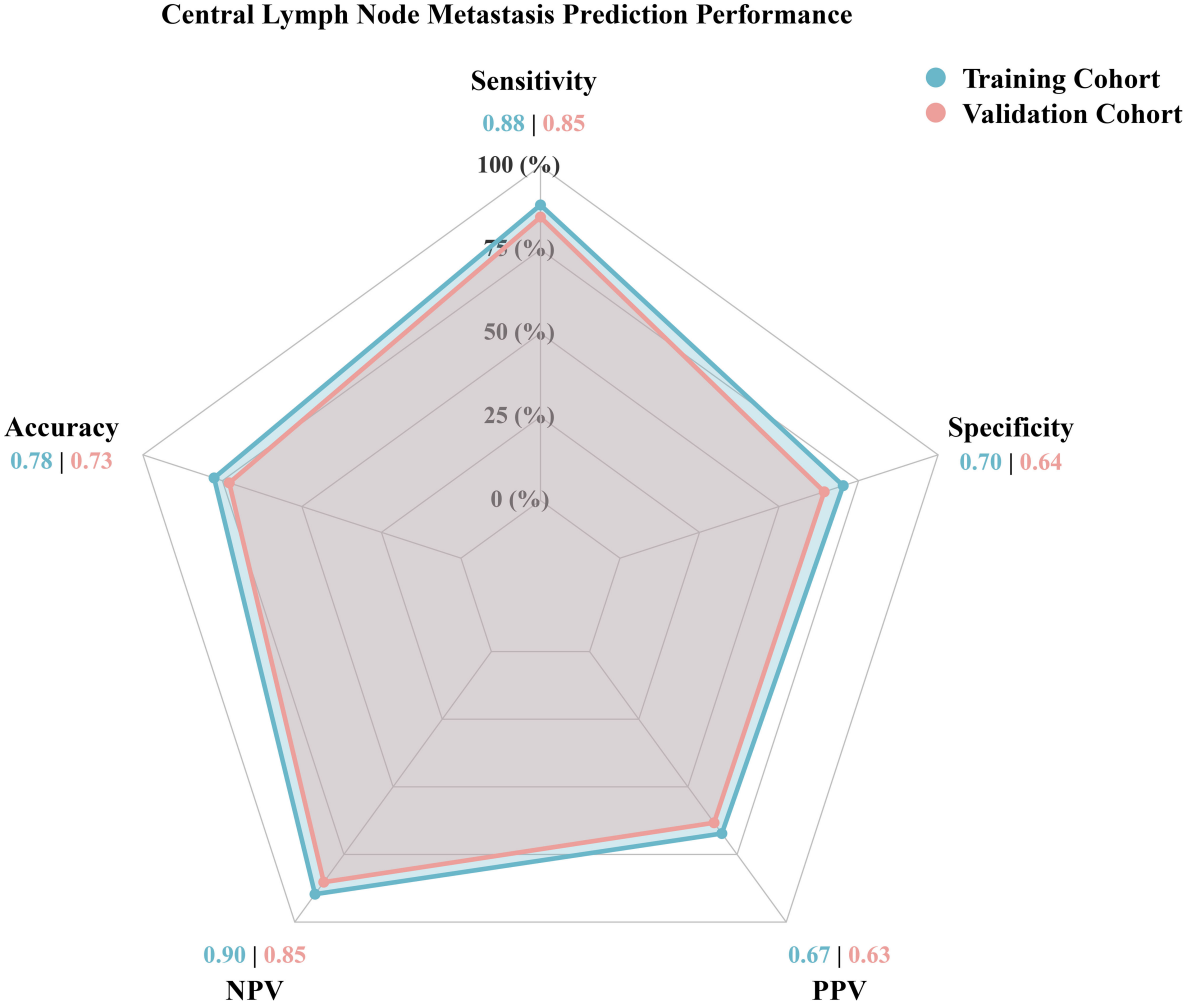
Performance metrics of the new model in the training and testing cohorts.

The new model exhibited high sensitivity and negative predictive value (NPV) in both cohorts (**Table 3**, **Figure 6**). In the training cohort, the sensitivity was 88.4% (95% CI: 81.6% - 95.1%), specificity 70.2% (95% CI: 62.1% - 78.2%), PPV 67.3% (95% CI: 58.6% - 75.9%), NPV 89.7% (95% CI: 83.6% - 95.7%), and accuracy 77.6% (95% CI: 72.0% - 83.3%). In the testing cohort, the sensitivity was 84.7% (95% CI: 75.6% - 93.9%), specificity 64.2% (95% CI: 53.8% - 74.6%), PPV 63.3% (95% CI: 52.7% - 73.9%), NPV 85.2% (95% CI: 76.3% - 94.1%), and accuracy 72.9% (95% CI: 65.5% - 80.2%). The consistently high NPV across both cohorts indicates the model’s strong capability to rule out CLNM.

**Table 3:** Performance Metrics of the new model.

Metric	Training cohort	95% CI	Testing cohort	95% CI
Sensitivity	88.4%	81.6-95.1	84.7%	75.6-93.9
Specificity	70.2%	62.1-78.2	64.2%	53.8-74.6
PPV	67.3%	58.6-75.9	63.3%	52.7-73.9
NPV	89.7%	83.6-95.7	85.2%	76.3-94.1
Accuracy	77.6%	72.0-83.3	72.9%	65.5-80.2

*CI, confidence interval; PPV, positive predictive value; NPV, negative predictive value.

### Comparison between the new prediction model and the clinicopathological prediction model

**Figure 7:**
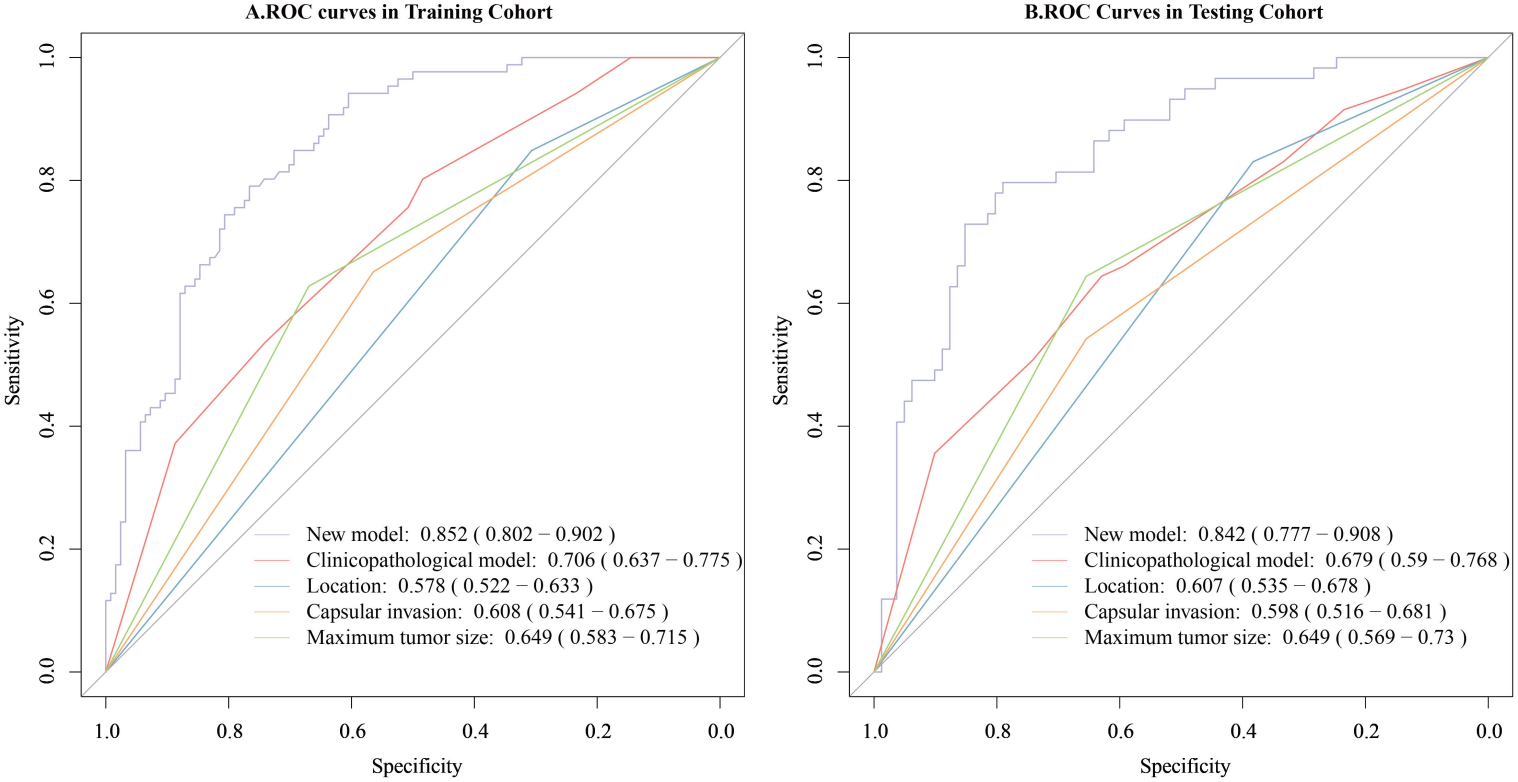
Comparison of the predictive value for CLNM of the new model and the clinicopathological model. **(A)** Comparison of the predictive value of the two models for CLNM in the training cohort. **(B)** Comparison of the predictive value of the two models for CLNM in the testing cohort. The new model combined the collagen signature and clinicopathological risk factors and the clinicopathological model included only clinicopathological risk factors.

The new model was compared with the clinicopathological model. The clinicopathological prediction model included the parameters that were statistically important in the multivariate logistic regression analysis, i.e., tumor size, tumor location and TCI. In the training cohort, the new model outperformed the clinicopathological model, with AUCs of 0.852 (95% CI, 0.802–0.902) versus 0.706 (95% CI, 0.637–0.775), respectively. The difference was statistically significant (DeLong’s test, p < 0.001) (**Figure 7A**). Similarly, in the testing cohort, the new model demonstrated superior discrimination, with AUCs of 0.842 (95% CI, 0.777–0.908) versus 0.679 (95% CI, 0.590–0.777) for the clinicopathological model (DeLong’s test, p < 0.001) (**Figure 7B**).

### Subset analysis comparing BRAF mutation and ultrasound features with new model

In a subset of 120 patients with available molecular and ultrasound data, the BRAF V600E mutation was detected in 67 patients (55.8%). Among ultrasound characteristics, microcalcification (OR = 2.368, 95% CI 1.092–5.340, p = 0.032) and ill-defined margins (OR = 2.579, 95% CI 1.216–5.580, p = 0.014) were significantly associated with CLNM (**Supplementary Table 2**). These two variables were incorporated into an ultrasound radiomics model. The new model maintained superior predictive performance (AUC = 0.860) compared with BRAF mutation (AUC = 0.618, DeLong’s test p <0.001) or ultrasound radiomics model (AUC = 0.664, DeLong’s test p=0.001). (**Supplementary Figure 2**).

## Discussion

The existing clinicopathological risk factors lack sufficient accuracy for identifying CLNM in patients with thyroid cancer. To address this gap, we conducted a retrospective analysis of 350 PTC cases and developed a collagen signature derived from five SHG-based features. The collagen signature showed robust and independent predictive power and significantly outperformed the clinicopathological model alone. These findings highlight the utility of collagen remodeling as a structural biomarker that complements existing clinical and molecular indicators for individualized risk assessment.

Extracellular matrix (ECM) remodeling represents a central component of TME reprogramming and is a key determinant of invasive behavior in PTC (30, 31). The five SHG-derived collagen features selected in our model capture distinct TME-derived stromal phenotypes that are biologically integral to metastatic progression and their directions of association are consistent with known ECM biology. Collagen crosslink density, which showed a positive association with CLNM, reflects LOX-mediated stiffening driven by BRAF–TGF-β1 signaling (32, 33). Increased crosslinking enhances tensile strength and promotes force transmission, enabling directional migration toward lymphatic vessels (34). In contrast, collagen arrangement demonstrated a negative coefficient, indicating that highly uniform and orderly fiber patterns are associated with lower metastatic risk. This is consistent with observations that invasive tumors often lose microdomain-level alignment and exhibit fragmented, reoriented, or partially disrupted collagen architectures, which permit more adaptable and plastic modes of invasion. Similarly, the mean collagen intensity was negatively associated with CLNM, supporting the concept that metastatic potential is not determined by bulk collagen abundance, but rather by its biomechanical organization; densely fibrotic yet structurally stable matrices often correspond to less aggressive phenotypes. The negative association of GLCM-contrast aligns with the known decrease in local textural sharpness at invasive fronts, where protease-mediated ECM degradation produces smoother SHG gradients. Finally, Gabor-variance, positively associated with CLNM, quantifies multi-scale orientation dispersion and the dynamic ECM rearrangements characteristic of active metastatic niches (34). Together, these features reflect complementary dimensions of ECM mechanics, including stiffness, microdomain disruption, alignment loss, diminished local contrast, and increased remodeling complexity, and these supports their biological relevance as microenvironmental markers of lymphatic invasion.

Preoperative ultrasound has limited sensitivity in accurately assessing the deep anatomical spaces of the central neck compartment. In our study, we observed a CLNM rate of 42.3% in cases where preoperative examinations did not reveal any evidence of lymph node metastasis, which aligns with existing literature reporting CLNM rates ranging from 30% to 52.3% (35–37). Molecular predictors such as BRAF V600E mutations or circRNA panels, though informative, are costly and not universally available (10, 37, 38). Several deep learning models have shown promising potential in the radiomics of ultrasound and computed tomography, though further validation is still needed (39, 40). Compared to ultrasound radiomics models reporting AUCs of 0.70-0.85 (39), our collagen signature achieved comparable discriminative capacity (AUC = 0.852) while avoiding reliance on operator-dependent imaging protocols. Subset analysis further demonstrated that the new model outperformed BRAF mutation status and the ultrasound radiomics model, underscoring its complementary value. While genetic tests provide mechanistic insights and the clinicopathological factors including tumor size, capsular invasion, and location represent clinical manifestations of local aggressiveness and lymphatic spread potential, the collagen signature reflects tumor microenvironment remodeling. Their complementary inclusion enhances biological plausibility of the predictive model.

This study represents the first successful application of the multiphoton microscopy imaging system for predicting CLNM in PTC. The imaging system used in our research has a well-established track record and has been extensively validated in prior studies for its effectiveness in various biomedical applications (13, 19, 20). The SHG imaging protocol enabled real-time collagen analysis within 10 minutes per specimen, compatible with intraoperative frozen section workflows. This proof-of-concept study was conducted on FFPE tissues rather than frozen sections; thus intraoperative application remains technically challenging. However, rapid postoperative implementation is feasible, providing timely information to guide decisions on completion or prophylactic central neck dissection. Future optimization of real-time SHG imaging and automated feature extraction could enable intraoperative decision-making in routine surgical workflows.

This retrospective single-center study inevitably carries potential selection bias, and the internal random split does not fully substitute for external validation. CLNM rates could vary by iodine intake, ethnicity, and pathological practices, and our patient population may not represent global PTC heterogeneity. Future studies will incorporate multicenter external cohorts and predefined sample size calculations based on events-per-variable principles to confirm generalizability and predictive stability. As molecular data and ultrasound features were not all available for this retrospective cohort, future prospective studies will incorporate molecular, transcriptomic and ultrasound data to complement and validate the new model. Mechanistic validation through immunohistochemical or transcriptomic correlation with CAF or LOX markers would further strengthen the biological interpretation of the collagen signature.

## Conclusions

This study establishes an integrative predictive framework that synergizes tumor microenvironment collagen signatures with key clinicopathological variables (tumor location, size, and capsular invasion) to refine risk stratification of CLNM in papillary thyroid carcinoma. Future implementation of this model could standardize surgical decision-making and optimize postoperative surveillance protocols.

## Data Availability

The raw data supporting the conclusions of this article will be made available by the authors, without undue reservation.
