# Supplementary material for: Development and validation of a collagen signature to predict central lymph node metastasis in papillary thyroid cancer
Source: Front Endocrinol (Lausanne). 2025 Nov 28;16:1691788. doi: 10.3389/fendo.2025.1691788 (PMC12698418; doi:10.3389/fendo.2025.1691788)

**SUPPLEMENTARY FORMULA 1**

Collagen signature=

0.188 * collagen crosslink density

-0.116 * collagen arrangement

-0.077* mean of collagen intensity

-0.030* contrast of 45° GLCM of the collagen fibers at direction 2

+0.118* variance convolution of Gabor filter images on scale 2 at direction 5

| **SUPPLEMENTARY TABLE 1.** Univariate Logistic regression analysis of the association of the collagen signature and clinical characteristics with CLNM in the training and testing cohort | | | | |
| --- | --- | --- | --- | --- |
| Variables | Training cohort | | Testing cohort | |
|  | OR (95% CI) | *p* | OR (95% CI) | *p* |
| Collagen signature | 5.201(3.141-8.611) | <0.001 | 4.737(2.399-9.351) | <0.001 |
| Age (years) (≤55 vs ˃55) | 0.637(0.314-1.292) | 0.637 | 0.863(0.370-2.010) | 0.732 |
| Gender (male vs female) | 0.720(0.410-1.261) | 0.250 | 0.774(0.393-1.526) | 0.459 |
| BMI (kg/m²) (≤23 vs ˃23) | 1.096(0.629-1.910) | 0.746 | 1.150(0.587-2.251) | 0.684 |
| Multifocality (negative vs. positive) | 1.126(0.595-2.132) | 0.715 | 1.235(0.579-2.631) | 0.585 |
| Location (middle-inferior portion vs. upper) | 0.403(0.200-0.814) | 0.011 | 0.390(0.176-0.863) | 0.020 |
| Maximum tumor size(cm) (≤1 vs ˃1) | 3.416(1.921-6.074) | <0.001 | 2.397(1.206-4.765) | 0.013 |
| Thyroid capsular invasion (negative vs. positive) | 2.420(1.371-4.270) | 0.002 | 2.243(1.129-4.459) | 0.021 |

**SUPPLEMENTARY TABLE 2.** Univariate and Multivariate Logistic Regression Analysis of Ultrasound Features with CLNM in the Subset

| Variable | Univariate OR (95% CI) | *p* | Multivariate OR (95% CI) | *p* |
| --- | --- | --- | --- | --- |
| Microcalcification | 2.368 (1.092–5.340) | 0.032 | 2.852 (1.263–6.811) | 0.014 |
| Ill-defined Margin | 2.579 (1.216–5.580) | 0.014 | 3.046 (1.387–6.957) | 0.007 |
| Echogenicity | 1.877 (0.809–4.585) | 0.152 | — | — |
| ShapeRatio | 1.190 (0.571–2.504) | 0.644 | — | — |


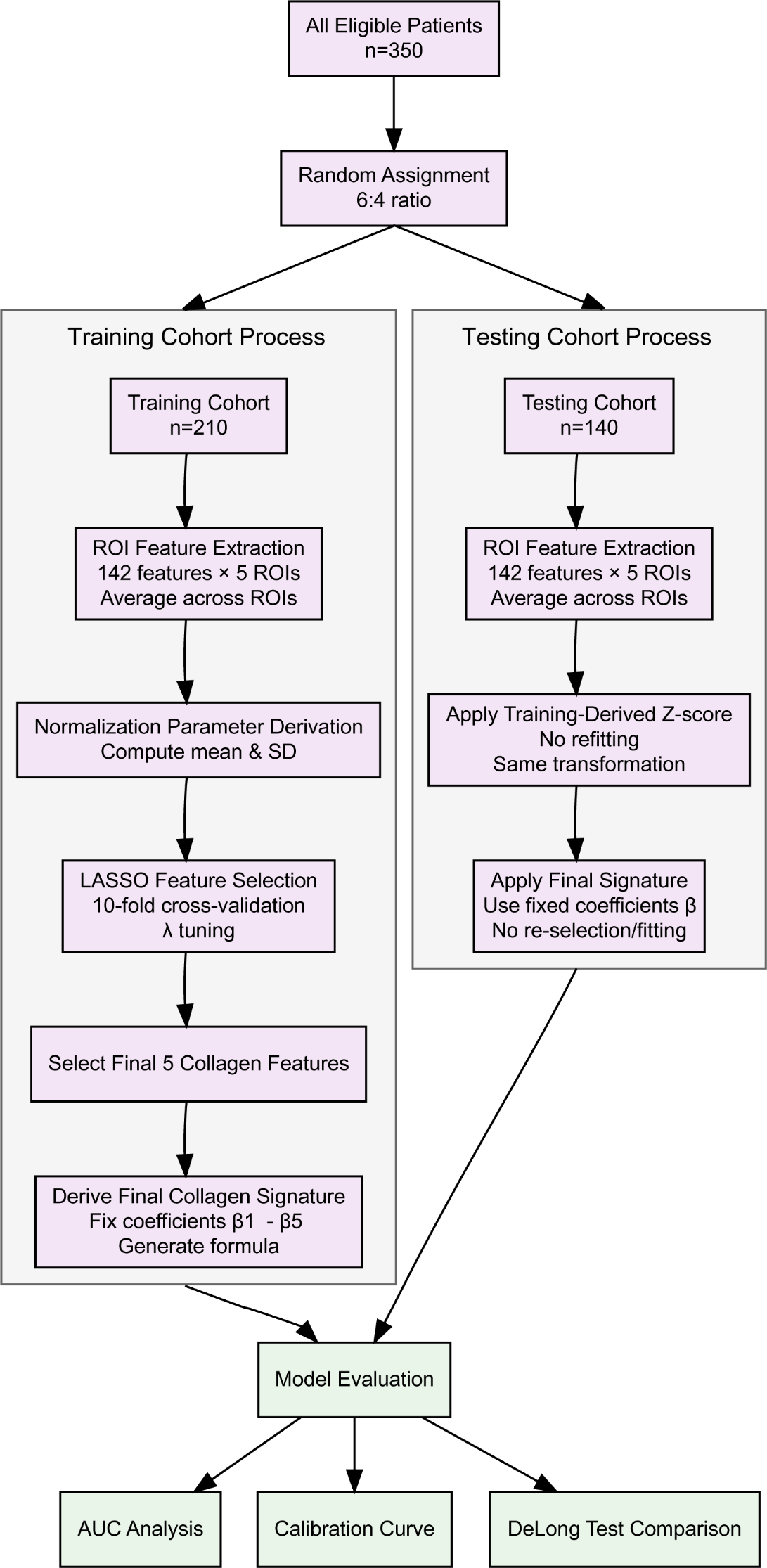
 **SUPPLEMENTARY FIGURE 1.** Workflow of The Training–Testing Pipeline to Avoid Data Leakage

**SUPPLEMENTARY FIGURE 2.** Comparison of The Predictive Value for CLNM of The New Model With BRAF and Ultrasound Radiomics Model in The Subset


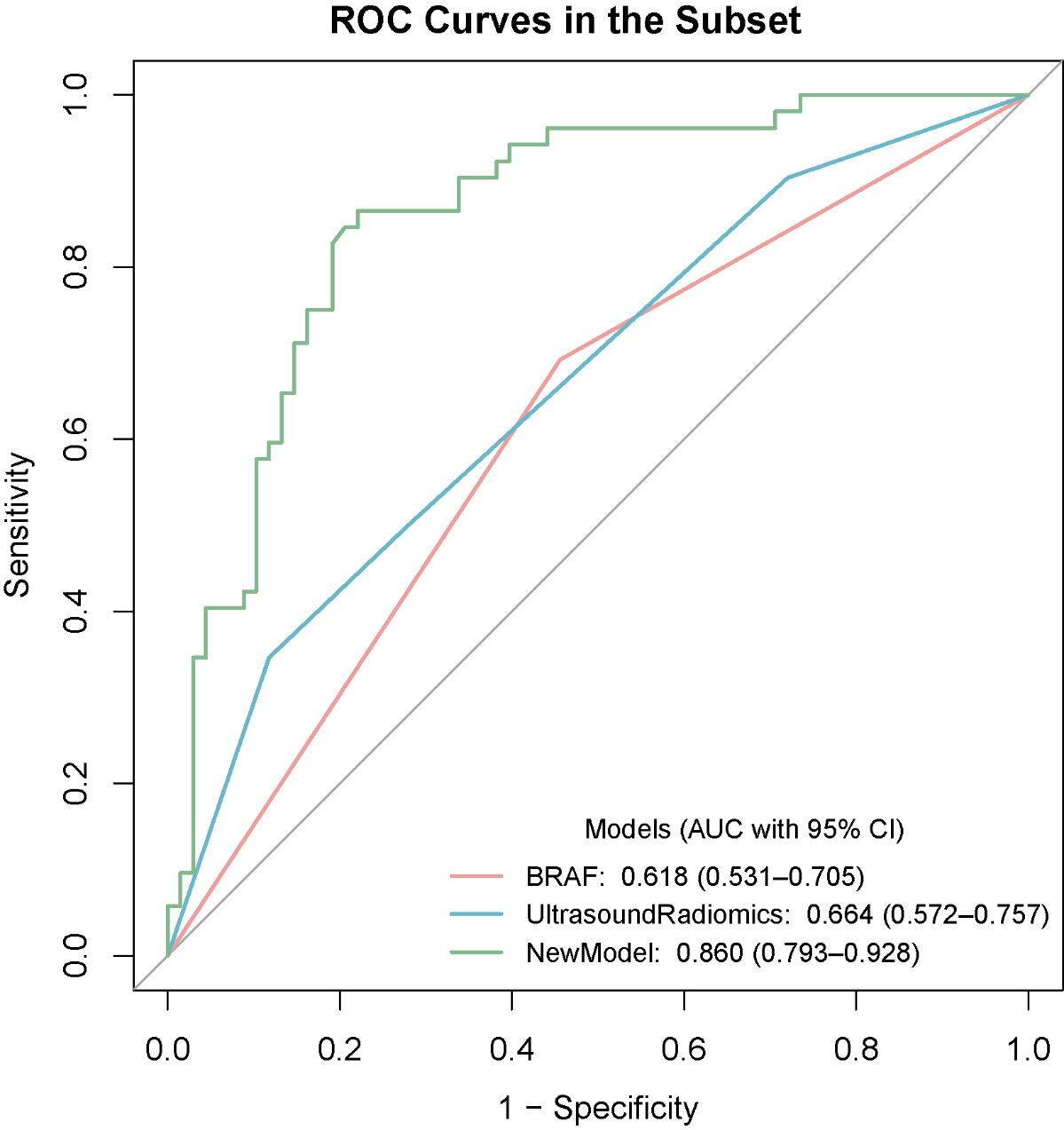

Supplement: Supplementary file 1 [file DataSheet1.docx]
